# Identification of circulating microRNAs during the liver neoplastic process in a murine model of hereditary tyrosinemia type 1

**DOI:** 10.1038/srep27464

**Published:** 2016-06-10

**Authors:** Francesca Angileri, Geneviève Morrow, Jean-Yves Scoazec, Nicolas Gadot, Vincent Roy, Suli Huang, Tangchun Wu, Robert M. Tanguay

**Affiliations:** 1 Laboratoire de génétique cellulaire et développementale, IBIS and PROTEO, Département de biologie moléculaire, biochimie médicale et pathologie, Faculté de médecine, 1030 avenue de la Médecine, Université Laval, Québec, Canada, G1V 0A6; 2 Dept of Occupational and Environmental Health and Key Laboratory of Environment and Health, Ministry of Education and State Key Laboratory of Environmental Health (Incubating), School of Public Health, Tongji Medical College, Huazhong University of Science and Technology, Wuhan 430030, China; 3 Anipath, Faculté Laennec, rue Guillaume Paradin, 69372 Lyon Cedex 08, France; 4 Key Laboratory of Molecular Biology of Shenzhen, Center for Disease Control and Prevention, Shenzhen, 518055, Guangdong, China

## Abstract

Hereditary tyrosinemia type 1 (HT1) is a severe inborn error of metabolism, impacting the tyrosine catabolic pathway with a high incidence of hepatocellular carcinoma (HCC). Using a HT1 murine model, we investigated the changes in profiles of circulating and hepatic miRNAs. The aim was to determine if plasma miRNAs could be used as non-invasive markers of liver damage in HT1 progression. Plasma and liver miRNAome was determined by deep sequencing after HT1 phenotype was induced. Sequencing analysis revealed deregulation of several miRNAs including let-7/miR-98 family, miR-21 and miR-148a, during manifestation of liver pathology. Three miRNAs (miR-98, miR-200b, miR-409) presenting the highest plasmatic variations among miRNAs found in both plasma and liver and with >1000 reads in at least one plasma sample, were further validated by RT-qPCR. Two of these miRNAs have protein targets involved in HT1 and significant changes in their circulating levels are detectable prior an increase in protein expression of alpha-fetoprotein, the current biomarker for HCC diagnosis. Future assessment of these miRNAs in HT1 patients and their association with liver neoplastic lesions might designate these molecules as potential biomarkers for monitoring HT1 damage progression, improving diagnosis for early HCC detection and the design of novel therapeutic targets.

Hereditary tyrosinemia type 1 (HT1, OMIM 276700) is a rare inherited metabolic disease associated with a high risk of liver cancer development and usually fatal before two years of age if not treated effectively (reviewed in^1^). HT1 is caused by a deficiency in fumarylacetoacetate hydrolase (FAH, EC 3.7.1.2), the last enzyme of the tyrosine catabolic pathway^2^. Three main clinical forms of HT1 have been described, each associated with liver and kidney failures^3^. The combination of NTBC (2-(2-nitro-trifluoromethylbenzoyl)-1,3-cyclohexanedione) intake with a low-tyrosine diet, and liver transplantation for the most severe cases, represent the only treatments available for this disease^4^. However although this regimen prevents liver and kidney dysfunction in patients^5^, affected individuals can still develop several chronic complications including cirrhosis with a high risk of HCC^6^,^7^. HT1 patients are therefore closely followed by regular liver imaging and marker measurements such as alpha-fetoprotein (AFP) level^6^. However, patients monitoring is not completely effective since almost 80% of small HCCs foci do not show increase in AFP levels and liver image sensitivity is not effective in detecting small liver lesions^8^,^9^. For instance, HCC has been found on an explanted liver from a patient for whom the AFP level had not obviously increased and imaging had missed the detection^10^. Therefore additional predictive value from other parameters for early detection of tumorigenic process would help in the follow-up.

Growing evidence suggests that microRNAs (miRNAs) are deregulated in liver pathologies^11^. MiRNAs constitute an abundant class of short non-coding RNAs of approximately 18–24 nucleotides (nts) in length, which have key roles in cell development and differentiation by mediating the post-transcriptional regulation of protein-coding genes^12^. Circulating miRNAs have been reported to be associated with the physiological/pathological state of the tissue they are derived from, representing a blood-based fingerprint of the affected tissue^13^. Currently, high–throughput technologies have revealed comprehensive profiling of circulating miRNAs in a wide variety of cancers, driving the discovery of novel cancer pathways regulated by oncogenic or tumour suppressive miRNAs^14^. MiRNAs are not only found in solid tumours, but also in circulation and body fluids^15^, in a highly stable, cell-free form^16^. Since miRNAs expression is tissue-, tumour-, and disease-specific, they are considered a class of circulating nucleic acids useful as clinical biomarkers for cancer diagnosis^13^.

Here we examined the genome-wide expression profile of miRNAs in the *fah*^*−/−*^ mouse^17^ after induction of the HT1 phenotype, in order to evaluate the potential of using circulating miRNAs as a non-invasive method to assess HT1 liver damage progression.

## Results

### HT1 phenotype in *fah* ^*−/−*^ mouse

**Figure 1:**
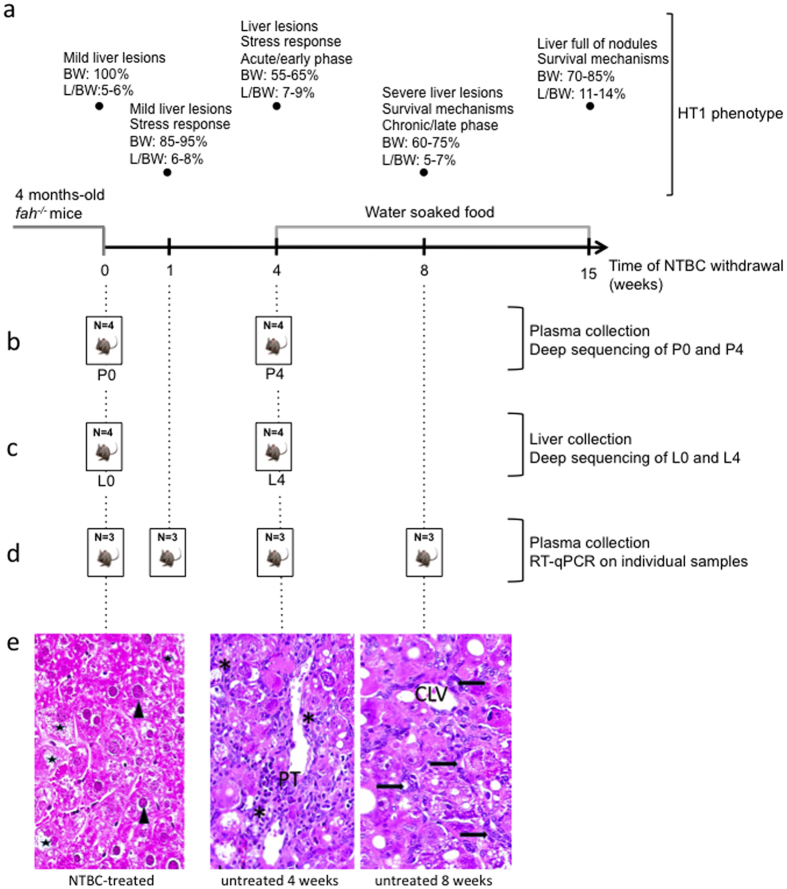
HT1 mouse model and study design. (**a)** *Fah*^*−/−*^ male mice are kept on NTBC diet until 4 months of age and then NTBC is withdrawn to allow HT1 progression. From the fourth week of NTBC withdrawal, water soaked food is added to the cages. The main characteristics of the chosen time points are presented to give an overview of HT1 disease progression^18^,^19^. BW: expected percentage of body weight comparatively to the weight at the beginning of experimentation. L/BW: expected percentage of liver/body weight ratio. (**b)** Deep sequencing of plasma miRNAs. Total RNAs were isolated from plasma of 4 mice treated with NTBC and equimolar amounts of total RNA extracts were pooled together to give P0. The same approach was used for 4 different mice removed from NTBC therapy during 4 weeks (P4). (**c)** Deep sequencing of liver miRNAs. Total RNAs were isolated from liver of 4 mice before NTBC interruption and equimolar amounts of total RNA extracts were pooled together to give L0. The same approach was used for 4 different mice untreated with NTBC for 4 weeks (L4). (**d)** RT-qPCR analysis. Total RNAs were isolated from three mice per time point. RT-qPCR analysis was performed on each mouse individually. (**e)** Representative liver sections of *fah*^*−/−*^ mice at different time point of the disease (HES staining). 1- *Fah*^*−/−*^ treated mice present mild hepatocellular changes due to the genetic background: hepatocytes are enlarged and often contain micro or macro-vesicular steatosis (black stars); large dysmorphic nuclei (arrowheads) show several well formed nucleoli (x350). 2–4 weeks off NTBC *fah*^*−/−*^ mice present liver lesions with hepatocellular changes and inflammation. The portal tract (PT) is enlarged; numerous inflammatory cells (*) are visible within the portal tract and into the adjacent lobule (x200). 3–8 weeks off NTBC *fah*^*−/−*^ mice present severe liver changes; hepatocyte plates are disorganized and thickened; numerous oval-like cells are present, arranged either in small sheets or in pseudoglandular structures (arrows). CLV: centrilobular vein (x250). Panels are representative of at least three pictures taken per sample of a total number of n = 4 mice for each series.

Due to the rarity and severity of HT1, studies addressing the basis and progression of the disease are usually performed in the *fah*^*−/−*^ mouse model. This model is very well characterized and reconstitutes many features of human HT1. Figure 1a shows a typical mouse protocol to study HT1; mice are put on an NTBC diet until 4 months of age and NTBC is then removed to allow HT1 progression. At the molecular level, the course of HT1 can be divided in two stages. The early stage is characterized by the activation of the stress response due to the accumulation of toxic metabolites and the late stage is characterized by the activation of cell death resistance and proliferation mechanisms with a robust induction of AFP^18^,^19^.

At the tissue level, liver damages are visible one week after NTBC withdrawal and neoplastic changes are observed in 100% of mice after 15 weeks of drug withdrawal^19^. Figure 1e shows representative liver sections of *fah*^*−/−*^ mice treated with NTBC and withdrawn from NTBC diet for 4 and 8 weeks. After 4 weeks of NTBC interruption, portal tracts are expanded by periportal fibrosis; numerous inflammatory cells are present in portal tracts and within the hepatic lobules. Hepatocellular changes include ballooning and micro- and macro-vesicular steatosis; some apoptotic cells are present (Fig. 1e). More severe histological lesions are observed after 8 weeks of NTBC withdrawal; architectural changes are characterized by the disorganization and thickening of hepatocyte plates; fibrosis and sinusoidal capillarization are present. Numerous oval-like cells are observed; these are morphologically similar to the oval cells described at the initiation of the process of chemical carcinogenesis, and are arranged in cohesive sheets or in pseudoglandular structures (Fig. 1e).

### Deep sequencing of small RNAs in plasma and livers from control and HT1 mice

Our initial aim was to determine plasma and liver miRNAs profiles of *fah*^*−/−*^ mice and see if there was any correlation between variations of miRNAs and progression of HT1. For this purpose, we performed deep sequencing of miRNAs from both tissues of NTBC-treated and 4 weeks NTBC-withdrawn *fah*^*−/−*^ mice (Fig. 1b,c). This withdrawal period was chosen based on previous studies demonstrating a peak in expression of stress proteins and the presence of liver lesions^18^,^19^ (Fig. 1a,e). Four mice were taken at each time point and total RNAs were submitted to LC Science (Houston, TX, USA) for processing and analysis. After quality control and samples assessment, 4 pools were generated by LC Science following our instructions and submitted to deep-sequencing analysis, *i.e* plasma NTBC-treated (P0), plasma 4 weeks NTBC-withdrawn (P4), liver NTBC-treated (L0) and liver 4 weeks NTBC-withdrawn (L4) (Fig. 1b,c). The pooling strategy was used here because the goal of the experiment was to have a general idea of the plasma and liver miRNA profiles rather than having data specific to some individuals.

From liver and plasma samples we detected a total of 1,002 mature miRNAs registered as mouse specific in miRbase (Release 20), and specifically 805 mature miRNAs in mouse plasma and 920 mature miRNAs in mouse liver (in both control and NTBC-withdrawn *fah*^*−/−*^ mice), with no significant difference within 5′ and 3′ end terminus (Supplementary Table S1). Among these, only a small fraction (4.6%) was expressed at very high levels (≥10,000 raw reads). The remaining miRNAs were expressed at relatively high (8.0%, 1000–9999 raw reads), moderate (15.5%, 100–999 raw reads), and low (71.7%, 1–99 raw reads) level.

### Therapy interruption induces changes in the expression of miRNAs in liver and plasma of *fah* ^*−/−*^ mice

For the following comparison analysis, the number of read copies from each miRNA tracked during mapping, was normalized to adjust for varying sequencing depth between the samples by dividing the counts by a library size parameter (Supplementary Table S1). This procedure yielded a total of 805 plasma miRNAs and 916 liver miRNAs for subsequent analysis.

The changes in miRNAs profiles due to the onset of HT1 were similar in plasma and liver (Table 1). Indeed, 7–9% were present only before HT1 induction (P0 and L0), 24–25% were present only once HT1 was triggered (P4 and L4) and 69% of the miRNAs were present in both types of samples (P0 and P4, L0 and L4). Another trend observed in both plasma and liver samples with the onset of HT1, was a decrease of 33% in the total number of reads (P0 vs P4, L0 vs L4). Interestingly, 72.1% of the mouse mature miRNAs were present both in plasma and liver (Supplementary Table S1). Mouse miR-192 showed the highest expression level in plasma of NTBC-treated mice (P0), whereas miR-21a was highest in the plasma of mice after disease onset (P4) (Table 1). Analyses of liver samples revealed miR-122 as the most abundant in both L0 and L4 samples. While this profiling does not document the influence of NTBC itself on miRNAs expression, the fact that mouse on NTBC diet develop normally during the time course of our standard protocol and do not show any major difference with wild-type mice except some mild liver lesions^19^ suggests that the effect of NTBC on the miRNAs profiles observed must be minor compared to the effect of HT1. The miRNAs changes associated with the progression of HT1 during the treatment were also considered as minor for the same reasons.

### The top deregulated plasma miRNAs are associated with pathological processes

**Figure 2:**
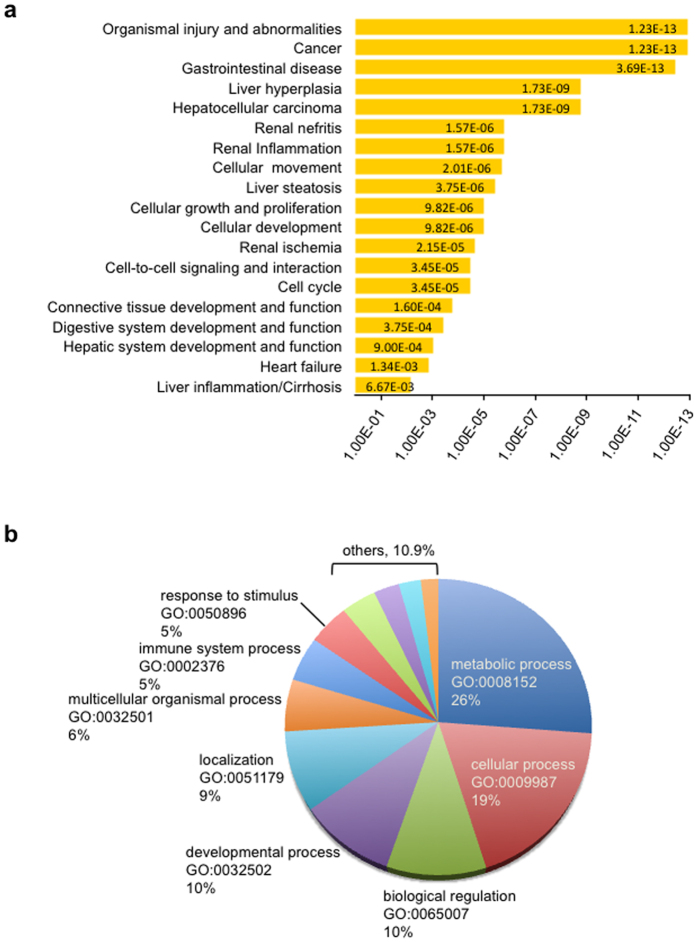
Pathway enrichment analysis and Gene Ontology (GO) classification of predicted miRNAs targets. (**a)** Functional analysis of predicted miRNAs targets was performed using Ingenuity software and integrated database. The *p*-value associated with a biological process or pathway annotation was calculated with the right-tailed Fisher’s Exact Test by the Ingenuity Function and Pathways analysis system. Top related pathways were those involved in organismal injury and abnormalities, cancer, gastrointestinal disease, liver hyperplasia and hepatocellular carcinoma. (**b)** Gene Ontology (GO) terms classification performed by DAVID bioinformatics software v 6.7. Top enriched processes were those involved in metabolic and cellular processes (26% and 19% respectively), biological process (10%), developmental process (10%) and localization (9%). P-values were obtained with the overrepresentation Fisher Exact Test and corrected by the Benjamini Hochberg post-hoc method, calculated by the EASE method in DAVID.

We then focused on miRNAs present in both plasma and liver to detect plasmatic variations likely related to the HT1 liver pathology (721 mouse miRNAs). Like many high-throughput experiments, a >1.5-fold change in reads count difference was adopted as a selection criterion for differentially expressed miRNAs between plasma sample of HT1 (P4) and control mice (P0) (304 mouse miRNAs, Supplementary Table S2). From these criteria, 207 miRNAs were found to be up-regulated in plasma following HT1 onset while 97 were down-regulated. Out of these 304 miRNAs, 125 did not show meaningful changes in the liver while 139 were deregulated in the same way in both plasma and liver. Interestingly, among the 16 miRNAs having >1000 normalized reads in at least one plasma sample (Table 2), several miRNAs (*e.g.* let-7/miR98 family members, miR-200b, miR-21a, miR-142, miR-192, miR-148a) have already been reported for their implication in liver carcinogenesis and other pathological conditions^13^,^20^. Accordingly, target prediction of these 16 plasma miRNAs has identified proteins affecting mainly hepatic and renal systems and involved in critical biological processes such as organismal injury, cancer, cellular growth and proliferation (Fig. 2a). Moreover, gene ontology terms classification of the potential miRNAs targets^21^, revealed enrichment in important biological processes like metabolic regulation, apoptosis, immune system process, cell-to-cell communication, protein transport and localization (Fig. 2b). Together these results suggest a general breakdown of tissue integrity and an increase in immune responses and tissue repairing processes after NTBC interruption in *fah*^*−/−*^ mice suffering of progressive liver failure.

### Validation of changes in circulating miRNAs levels during HT1 progression

To confirm the modulation of specific miRNAs during progression of the HT1-induced liver pathology in *fah*^*−/−*^ mice after NTBC removal, three miRNAs (namely miR-200b-3p, miR-98-5p and miR-409-5p) were selected for validation by RT-qPCR (Table 2, highlighted miRNAs). These miRNAs were chosen because they were found in both plasma and liver samples, in addition to have at least 1000 reads in one group of plasma sample and >1.5 Log_2_ fold change between NTBC-treated (P0) and –untreated (P4) animals.

**Figure 3:**
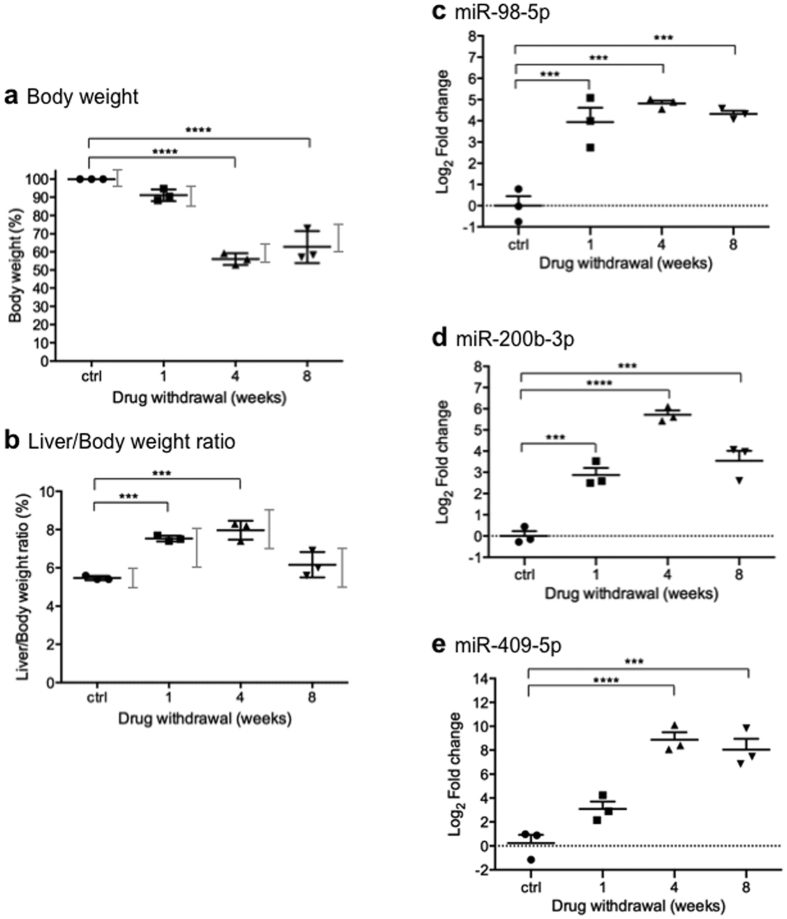
Modulation of plasma levels of miR-98, miR-200b and miR-409 after NTBC withdrawal. (**a–e)** Mice were weighted three times per week and periodically examined for signs of clinical illness during the protocol. (**a)** Graph represents the percentage of body weight of each mouse at the indicated time point comparatively to its weight at the beginning of experimentation. (**b)** Graph represents the percentage of liver/body weight ratio of each mouse at the indicated time point. Grey bars represents the values expected for each time point (Fig. 1a) and are based on previous experiments^19^. (**c–e)** RT-qPCR analysis of miR-98, miR-200b and miR-409 plasma levels in *fah*^*−/−*^ mice NTBC-treated and -untreated. *C. elegans* miR-39 was used as a loading control. Graph represents 3 individual per time point and median levels of miR-98-5p (**c**), miR-200b-3p (**d**) and miR-409-5p (**e**) in healthy controls and in NTBC-withdrawn mice (*i.e.* 1 week off, 4 weeks off and 8 weeks off). The relative expressions of selected miRNAs were normalized to *C. elegans* miR-39. Data are representative of at least three independent experiments. (**a–e)** Statistical significance was assessed by ordinary one way ANOVA followed by the Dunnett’s post hoc multiple comparisons test. *P*-values are represented by asterisk. ***p < 0.001 and ****p < 0.0001.

Since the aim of the validation protocol was to verify the level of the selected miRNAs at different stages of the disease, a new mouse protocol based on previous studies was undertaken with 4 different time points^18^,^19^; *i.e.* 0, 1, 4 and 8 weeks without NTBC (Fig. 1d). The 1 week withdrawal point was chosen to reveal if potential miRNAs biomarkers could detect the HT1 phenotype from the beginning of its pathological manifestations^19^, while the 4 and 8 weeks time points represented manifestation of acute and chronic phase respectively, with increasing risk of tumour development^19^ (Fig. 1a). NTBC-treated mice were used as reference sample. Importantly, all plasma samples were analysed individually. HT1 progression was carefully followed during the experiment. Figure 3a shows that the body weight of each mouse at a given drug withdrawal time point was in the expected range according to previous reports^19^ (Fig. 1a). Moreover, the liver/body weight ratio at the time of sacrifice was also consistent with previous findings^19^ (Figs 3b and 1a).

**Figure 4:**
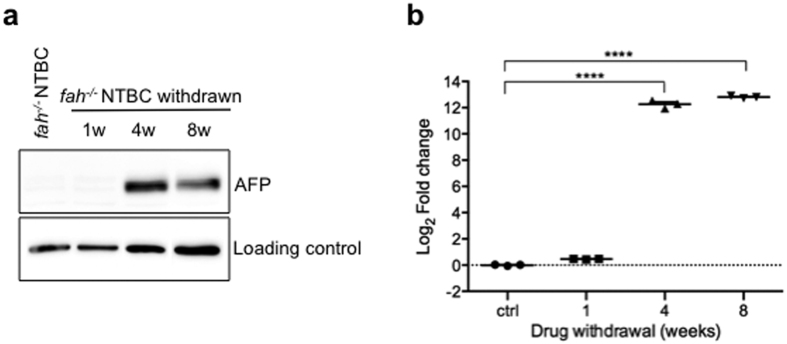
Time-course of AFP expression in liver during HT1 progression. (**a)** Representative immunoblot of AFP protein expression level in liver from individual mouse treated with NTBC or withdrawn from treatment for 1, 4 or 8 weeks. Western blots are representative of three independent experiments and were done on the livers from the same mice as the one used for RT-qPCR experiments. (**b)** Densitometric analysis of AFP expression. Values were normalized to loading control levels and finally compared to the *fah*^*−/−*^ healthy control (NTBC-treated). Data in graphs represents three mice for each time point. Statistical significance was assessed by ordinary one way ANOVA followed by the Dunnett’s post hoc multiple comparisons test. *P*-values are represented by asterisk: ****p < 0.0001.

Analysis of RT-qPCR results showed that the expression profile of the selected miRNAs was significantly different in NTBC-withdrawn and in NTBC-treated matched control mice (Fig. 3c–e). Specifically miR-98-5p and miR-200b-3p changed significantly from the early stage of HT1 manifestation (Fig. 3c,d). The miR-409-5p, despite a less significant variation in the first week of withdrawal (Fig. 3e), was the one showing the highest expression value after the long term therapy discontinuation, with almost the same expression value throughout acute and chronic phase response. For each of the miRNAs tested the highest fold change corresponded to the acute phase of the disease (4 weeks NTBC-withdrawal) (Fig. 3c–e). This could denote that the acute phase of HT1 disease, characterized by highest expression of stress proteins^18^, corresponds also to the highest miRNAs mobilization. However, this could also be due to the fact that these miRNAs were selected based on deep sequencing data at this particular time-point. Interestingly, miR-98-5p and miR200b-3p showed significant changes in circulating levels prior to AFP protein increase in liver, revealing a potential use as diagnostic tools (Figs. 3c,d and 4).

## Discussion

Deep sequencing profiling of liver and plasma miRNAome in the HT1 murine model during the pathogenic process following removal of the drug NTBC was performed. Sequencing results showed that miRNAs expression levels in plasma and liver of *fah*^*−/−*^ mice were distributed over 4 orders of magnitude and a significant number of miRNAs were either not expressed or expressed at very low levels. Several miRNAs presented variation in sequences compared to the annotated molecules (*i.e.* isomiRs), indicating a dynamic and versatile miRNAome in HT1.

The onset of HT1 pathology due to NTBC withdrawal resulted in a noticeable change in the amount of detectable miRNAs in both plasma and liver and in a significant variation of their relative expression levels. To associate the circulating signature with HT1 liver pathology, mouse miRNAs present in both plasma and liver were considered. Interestingly, we noticed overlap of several molecules with prominent roles in liver function. Among these, several miRNAs including let-7/miR-98 family, miR-21, miR-34a/c, miR-142, miR148a, and miR-192 predominantly expressed in liver, exhibited elevated changes in plasma samples^20^. Several additional miRNAs that have already been related to pathological conditions and neoplastic lesions were overrepresented among circulating molecules such as miR-155 (part of the miR-17-92 cluster)^22^, miR-200 family^23^, miR-148a^24^ and miR-375^25^.

Although some studies report of a low or even inverse correlation between miRNAs expression levels in tissues and plasma^20^, differential analysis of overlapping molecules showed a similar pattern for almost half of plasmatic and hepatic miRNAs (139/304). Differences were also observed and, among these, several members of the let-7 family were up-regulated in plasma after 4weeks of NTBC interruption despite an invariable expression in liver. As previously demonstrated, important changes in liver molecular pathways occur already after the first week of NTBC withdrawal^18^,^19^,^26^, and circulating levels of miRNAs might reflect these early changes representing potential indicator of the hepatic state during HT1 degenerative process.

The variation of miR-98, miR-200b and miR-409 plasmatic expression was validated at different time-point of HT1 disease. The pattern of HT1-induced up-regulation of miR-409 was different from the two other miRNAs, with significant variations starting at 4 weeks. This time point correlates with a significant increase of alkaline phosphatase (hepatocellular dysfunction) and γ-glutamyl-transferase (hepatocyte inflammation) in mice serum^26^. Some reports have shown a correlation between miR-409 overexpression and epithelial-mesenchymal transition and tumour growth in prostate cancer progression^27^,^28^, while others report an onco-suppressive action of miR-409 and demonstrate its down-regulation in gastric and lung cancers^29^,^30^. Although no functional study has been conducted to establish its role in liver injury progression, our result unveils for the first time an important deregulation of miR-409 circulating levels in the HT1 pathological process.

MiR-98 and miR-200b were shown to be up-regulated from the first week following HT1 onset a moment which corresponds to the previously reported drastic increase in alanine transaminase (hepatocyte inflammation) serum levels^26^. Interestingly, both of these miRNAs have targets that have already been shown to be associated with HT1 disease progression. Indeed, an indirect up-regulation of HO-1 gene expression by miR-98 action has been related with oxidative stress injury in hepatocytes^31^ and, similar to the induction of plasma miR-98 demonstrated here, HO-1 expression in liver was shown to increase in mice at the beginning of HT1 pathological process^19^,^32^. Similarly, miR-200b has been found positively correlated with liver fibrosis progression and PI3K/Akt pathway regulation^33^,^34^, mechanisms that are present in HT1 progression^19^,^26^. Since combined increased expression of miR-98 and miR-200b has also been connected with differentiation stage of cancer phenotype progression^23^, the high levels of these molecules in plasma of mice after NTBC removal (Fig. 3c,d), warrants further validation of these molecules as potential biomarkers of liver injury in HT1 patients. Especially since both miRNA were significantly up-regulated 1 week after-NTBC withdrawal, a time-point at which AFP protein was still almost not detectable in the liver.

In summary, this investigation provides evidence for deregulation of plasma and liver miRNA profiles during HT1 manifestation. Among the validated miRNAs affected by HT1, two are known to be involved in liver injury processes and can be linked to HT1 through their known targets. While it is premature to identify miR-98 and miR-200b as biomarkers of the state of liver injury, our results clearly warrant further investigation. Validation of such circulating biomarkers in plasma/serum of affected patients could potentially represent a new standard for a non-invasive routine clinical application in combination with pre-existing therapeutic and diagnostic methodologies.

## Materials and Methods

### Chemicals

NTBC (S. Lindstedt, Gothenburg University, Sweden) was diluted in water (7.5 mg/L) and the pH was adjusted at 7–7.3. Synthetic *Caenorhabditis elegans* miR-39 was from Qiagen (Mississauga, ON, Canada).

### Animal maintenance and treatment

Four months-old *fah* knockout (*fah*^Δ*exon5*^, referred to here as *fah*^*−/−*^) male mice^17^ were fed with a standard rodent chow (Charles River Rodent, Purina 5075-U.S, Agribrands, St-Hubert, QC, Canada) containing 0.51% tyrosine and 0.82% phenylalanine, and housed in a controlled environment with 12h light-dark cycles. Pregnant females and *fah*^*−/−*^pups received NTBC in drinking water until beginning of experimentation. To induce the HT1 phenotype, 4 months-old *fah*^*−/−*^ mice were withdrawn from NTBC therapy for periods of 1 to 8 weeks with our standard feeding protocol^18^,^19^ (Fig. 1a). Mice were weighted three times per week and periodically examined for signs of clinical illness. At the end of each starvation period, 3 to 4 mice were anesthetized by injection of ketamine-xylazine, and blood and livers were harvested and stored at −80 °C. *Fah*^*−/−*^ mice receiving NTBC *ad libitum* (n = 3–4) were used as healthy control. All animal experiments were performed according to the guidelines of the Canadian Council on Animal Care (CCAC). All experimental protocols were approved by the animal care committee of Université Laval (CPA-UL).

### RNA isolation

Isolation of the total RNAs from plasma and liver (200 μl and 0.26 mg respectively for each mouse) was performed using the mirVana PARIS miRNA Isolation Kit (Ambion 1556, Austin, TX). Due to the low amount of plasma available from each mouse and the low miRNAs concentration in biological fluids^16^, plasma samples were treated twice with acid-phenol chloroform and glycogen was included for a better RNA recovery. RNA quality and purity were verified on an Agilent 2100 Bioanalyzer system and Agilent RNA Nano 6000 LabChip kits (Agilent Technologies, Santa Clara, CA, USA). *C. elegans* miRNA cel-miR-39 was added into the denatured plasma samples to normalize sample-to-sample variation in the isolation step. The spiked-in miRNA was introduced after addition of 2X Denaturing Solution (Ambion) to the plasma sample to avoid degradation by endogenous plasma RNases.

### RNA deep sequencing and data analysis

For deep sequencing analysis, plasma and liver total RNA extracts were isolated from *fah*^*−/−*^ control mouse (NTBC-treated) and *fah*^*−/−*^ withdrawn from NTBC during 4 weeks (HT1 mice) (Fig. 1a–c). These two time points were chosen as representative of mice in healthy state (control) and mice suffering of acute pathophysiological manifestation of the disease (4 weeks after therapy interruption)^19^.

Total RNA extracts were submitted to LC Science (Huston, TX, USA) for further processing. Four pools, constituted each from total RNA from 4 different mice, were generated with equimolar RNA amounts by LC Science, following our instructions (*i.e.* plasma from control mice (P0), plasma from HT1 mice (P4), liver from control mice (L0) and liver from HT1 mice (L4)) (Fig. 1b,c). Each sample was processed to generate a cDNA library using the Illumina TruSeqTM Small RNA Preparation kit according to Illumina’s TruSeqTM Small RNA Sample Preparation Guide (see Supplementary File S1). The purified cDNA library was used for cluster generation on Illumina’s Cluster Station and then sequenced on Illumina GAIIx. Raw sequencing reads (40 nts) were obtained using Illumina’s Sequencing Control Studio software version 2.8 (SCS v2.8) following real time sequencing image analysis and base calling by Illumina’s Real-Time Analysis version 1.8.70 (RTA v1.8.70). The extracted sequencing reads were analysed using a proprietary pipeline script, ACGT101-miR v4.2 (LC Sciences), as described in the Supplementary material (Supplementary File S1).

### Quantitative reverse transcription polymerase chain reactions (RT-qPCRs)

Expression levels of three selected miRNAs were confirmed by RT-qPCR in plasma from 12 mice subjected to different length of therapy interruption (*i.e.* from 0 to 8 weeks NTBC withdrawal, n = 3/time-point). Importantly, in these experiments mice were treated individually. Due to the low and variable RNA content of plasma, a fixed volume of RNA eluate (5 μl) from a given volume of starting plasma, rather than a fixed mass of RNA, was used as input into the reverse transcription reactions^16^. *C. elegans* miRNA spiked-in during the RNA isolation process was used as internal reference for normalization of technical variations between samples. Input RNA was reverse transcribed following the manufacturer’s protocol of the TaqMan miRNA Reverse Transcription Kit (Applied BioSystems, Foster City, CA). RT products were diluted 1:5 and subjected to quantitative PCR (qPCR) in triplicate on a 7500 Fast Real-Time PCR System (Applied BioSystems, Foster City, CA). Data were analysed with 7500 Software version v2.3 (Applied BioSystems), with the automatic setting for assigning baseline and C_t_ (cycle threshold) determination. The miRNA expression levels were normalized against cel-miR-39 and calculated by the equation 2^–ΔCt^, in which ΔC_t_ = C_t_ miRNA–C_t_ cel-miR-39.

### Target gene prediction and enriched biological function analysis

The Ingenuity incorporated analysis of TarBase (http://diana.cslab.ece.ntua.gr/tarbase/)^35^, miRecords (http://mirecords.biolead.org/)^36^ and TargetScan (http://www.targetscan.org/)^37^, were used to predict the potential target genes of detected miRNAs. Gene Ontology (GO) and KEGG pathways enrichment analysis of these target genes were performed with DAVID Bioinformatics Resources 6.7 (http://david.abcc.ncifcrf.gov)^21^ and PANTHER classification system (http://pantherdb.org/)^38^.

### Histological Analysis

Mouse livers were fixed in 4% PBS-buffered paraformaldehyde, pH 7.4, and kept at 4 °C. Tissue samples were embedded in paraffin-wax at 58 °C. Four micrometer sections were prepared and stained with hematoxylin-eosin-saffron (HES) at ANIpath, a university platform for experimental animal histopathology (Lyon, France). Analysis and interpretation were done by a pathologist experienced in both human and experimental liver pathologies (JYS). Classification of liver changes was performed according to international recommendations^39^.

### SDS-polyacrylamide gel electrophoresis (SDS-PAGE) and western blot

Frozen livers were homogenized with a teflon pestle at 10% (W/V) in RIPA Buffer (20 mM Tris-HCl, pH 7.5; 150 mM NaCl, 1 mM Na_2_EDTA, 1 mM EGTA, 1% NP-40) containing protease and phosphatase inhibitor cocktails (cOmplete, Mini, EDTA-free; PhosSTOP; Roche Diagnostics, Indianapolis, USA). Liver extracts were then centrifuged at 10,000 g for 15 minutes at 4 °C and protein concentration was measured in the supernatant with the Bio-Rad Protein Assay (Bio-Rad Laboratories Inc., Hercules, CA). Proteins were analysed as described previously^19^. The antibody against AFP (1:10,000) was a gift from Dr. L. Bélanger (CRCHUQ, QC, Canada) and the antibody against GAPDH (#5174; 1:5000; loading control) was from Cell Signaling Technology (Danvers, MA, USA). Densitometric analysis on western blot bands was performed using ImageJ 1.47v software. Values were normalized to loading control levels and finally compared to the *fah*^*−/−*^ healthy control (NTBC-treated). Data in graphs represents three mice for each time point.

### Statistical analysis

The Student’s t-test or ANOVA was used to analyse the differences between samples. Values of *p* < 0.05 were considered statistically significant. RT-qPCR experiments were repeated at least twice with similar results. All experiments included triplicate samples for each treatment group. Statistical analyses were performed with GraphPad Prism software v6.0b (La Jolla, CA, USA).

## Additional Information

**How to cite this article**: Angileri, F. *et al*. Identification of circulating microRNAs during the liver neoplastic process in a murine model of hereditary tyrosinemia type 1. *Sci. Rep.* **6**, 27464; doi: 10.1038/srep27464 (2016).

## Supplementary Material

Supplementary Information

Supplementary Table

## Figures and Tables

**Table 1:** Similarity and differences of plasma and liver miRNA profiles before and after HT1 onset.

	Plasma (805 mmu-miR total)		Liver (916 mmu-miR total)	
	Control (P0)(NTBC-treated)	HT1 (P4)(NTBC-untreated)	Control (L0)(NTBC-treated)	HT1 (L4)(NTBC-untreated)
Specific mmu-miR^1^	60 (7%)	191 (24%)	59 (9%)	229 (25%)
Total number of reads^1^	2,916,233	1,767,043	3,020,177	1,956,385
Most abundant mmu-miR	miR-192-5p(333,335 reads^2^)	miR-21a-5p(120,886 reads^2^)	miR-122-5p(785,568 reads^2^)	miR-122-5p(253,398 reads^2^)

^1^mmu-miR found in only one of the two plasma or liver samples.

^2^Normalized reads (Supplementary Table S1).

**Table 2:** Most abundant plasma miRNAs (>1000 reads in P0 or P4) having a Log_2_ fold change greater than 1.5 or lesser than -1.5 and being also present in at least one liver sample.

miR_name	Normalized read counts in plasma^1^		Log_2_ fold change (NTBC-untreated/-treated)	
	NTBC-treated(P0)	NTBC-untreated(P4)	Plasma (P4/P0)	Liver (L4/L0)
**miR-200b-3p**	**666.53**	**3,139.24**	**2.24**	**1.95**
**miR-98-5p**	**365.38**	**1,655.84**	**2.18**	**—**
**miR-409-5p**	**282.60**	**1,173.17**	**2.05**	**4.34**
miR-136-3p	727.90	2,880.37	1.98	3.19
miR-21a-5p	31,374.05	120,886.48	1.95	—
miR-142-3p	339.69	1,129.17	1.73	—
let-7d-5p	3,100.25	9,470.69	1.61	—
let-7a-5p	1,997.27	5,736.78	1.52	—
miR-106b-3p	5,629.12	1,918.62	−1.55	—
miR-151-3p	8,680.60	2,670.18	−1.70	—
miR-10b-5p	105,854.24	32,047.06	−1.72	—
miR-423-5p	3,853.60	1,075.40	−1.84	—
miR-378c	3,928.77	958.90	−2.03	—
miR-486-5p	242,915.53	48,666.34	−2.32	—
miR-148a-3p	77,032.13	15,401.51	−2.32	−2.33
miR-192-5p	333,335.37	64,636.71	−2.37	−1.89

^1^Only miRNA present in both plasma and liver samples, having read counts >1000 in P0 or P4 and Log_2_ fold change greater than 1.5 or lesser than –1.5 are presented. See Supplementary Table S2 for all the miRNA found in both plasma and liver and having >1.5 Log_2_ fold change.

Values in bold font: miRNA selected for validation by RT-qPCR.

—: Log_2_ fold change considered as non-meaningful (<1.5 and >–1.5).

## References

[b1] Mitchell G. A., Grompe M., Lambert H. & Tanguay R. M. In The Metabolic and Molecular Bases of Inherited Diseases Vol. Volume II (eds Scriver C. R., Beaudet A. L., Sly W. S. J. & Valle D. ) Ch. 79, pp. 1777–1805 (McGrawHill, 2001).

[b2] Lindblad B., Lindstedt S. & Steen G. On the enzymic defects in hereditary tyrosinemia. Proc Natl Acad Sci USA 74, 4641–4645 (1977). 270706 10.1073/pnas.74.10.4641 PMC432003

[b3] Russo P. A., Mitchell G. A. & Tanguay R. M. Tyrosinemia: a review. Pediatr Dev Pathol 4, 212–221 (2001). 11370259 10.1007/s100240010146

[b4] Lindstedt S., Holme E., Lock E. A., Hjalmarson O. & Strandvik B. Treatment of hereditary tyrosinaemia type I by inhibition of 4-hydroxyphenylpyruvate dioxygenase. Lancet 340, 813–817, 0140-6736(92)92685-9 (1992). 1383656 10.1016/0140-6736(92)92685-9

[b5] Larochelle J. . Effect of nitisinone (NTBC) treatment on the clinical course of hepatorenal tyrosinemia in Quebec. Mol Genet Metab 107, 49–54, S1096-7192(12)00211-9 (2012). 22885033 10.1016/j.ymgme.2012.05.022

[b6] de Laet C. . Recommendations for the management of tyrosinaemia type 1. Orphanet J Rare Dis 8, 8, 1750-1172-8-8 (2013). 23311542 10.1186/1750-1172-8-8 PMC3558375

[b7] Mayorandan S. . Cross-sectional study of 168 patients with hepatorenal tyrosinaemia and implications for clinical practice. Orphanet J Rare Dis 9, 107, s13023-014-0107-7 (2014). 25081276 10.1186/s13023-014-0107-7 PMC4347563

[b8] Carr B. I., Pancoska P. & Branch R. A. Low alpha-fetoprotein hepatocellular carcinoma. J Gastroenterol Hepatol 25, 1543–1549, 10.1111/j.1440-1746.2010.06303.x (2010). 20796153

[b9] Schutte K., Schulz C., Link A. & Malfertheiner P. Current biomarkers for hepatocellular carcinoma: Surveillance, diagnosis and prediction of prognosis. World J Hepatol 7, 139–149, 10.4254/wjh.v7.i2.139 (2015). 25729470 PMC4342597

[b10] van Ginkel W. G. . Hepatocellular Carcinoma in Tyrosinemia Type 1 Without Clear Increase of AFP. Pediatrics 135, e749–752, 10.1542/peds.2014-1913 (2015). 25667247

[b11] Callegari E. . MicroRNAs in liver cancer: a model for investigating pathogenesis and novel therapeutic approaches. Cell Death Differ 22, 46–57, 10.1038/cdd.2014.136 (2015). 25190143 PMC4262781

[b12] Bartel D. P. MicroRNAs: genomics, biogenesis, mechanism, and function. Cell 116, 281–297 (2004). 14744438 10.1016/s0092-8674(04)00045-5

[b13] Schwarzenbach H., Nishida N., Calin G. A. & Pantel K. Clinical relevance of circulating cell-free microRNAs in cancer. Nat Rev Clin Oncol 11, 145–156, 10.1038/nrclinonc.2014.5 (2014). 24492836

[b14] Roderburg C. & Luedde T. Circulating microRNAs as markers of liver inflammation, fibrosis and cancer. J Hepatol 61, 1434–1437, 10.1016/j.jhep.2014.07.017 (2014). 25306489

[b15] Kosaka N., Iguchi H. & Ochiya T. Circulating microRNA in body fluid: a new potential biomarker for cancer diagnosis and prognosis. Cancer Sci 101, 2087–2092, 10.1111/j.1349-7006.2010.01650.x (2010). 20624164 PMC11159200

[b16] Mitchell P. S. . Circulating microRNAs as stable blood-based markers for cancer detection. Proc Natl Acad Sci USA 105, 10513–10518, 10.1073/pnas.0804549105 (2008). 18663219 PMC2492472

[b17] Grompe M. . Loss of fumarylacetoacetate hydrolase is responsible for the neonatal hepatic dysfunction phenotype of lethal albino mice. Genes Dev 7, 2298–2307 (1993). 8253378 10.1101/gad.7.12a.2298

[b18] Angileri F., Morrow G., Roy V., Orejuela D. & Tanguay R. M. Heat shock response associated with hepatocarcinogenesis in a murine model of hereditary tyrosinemia type I. Cancers (Basel) 6, 998–1019, cancers6020998 (2014). 24762634 10.3390/cancers6020998 PMC4074813

[b19] Angileri F. . Molecular changes associated with chronic liver damage and neoplastic lesions in a murine model of hereditary tyrosinemia type 1. Biochim Biophys Acta 1852, 2603–2617, 10.1016/j.bbadis.2015.09.002 (2015). 26360553

[b20] Wang K. . Circulating microRNAs, potential biomarkers for drug-induced liver injury. Proc Natl Acad Sci USA 106, 4402–4407, 10.1073/pnas.0813371106 (2009). 19246379 PMC2657429

[b21] Huang da W., Sherman B. T. & Lempicki R. A. Systematic and integrative analysis of large gene lists using DAVID bioinformatics resources. Nat Protoc 4, 44–57, nprot.2008.211 (2009). 19131956 10.1038/nprot.2008.211

[b22] Mendell J. T. miRiad roles for the miR-17-92 cluster in development and disease. Cell 133, 217–222, 10.1016/j.cell.2008.04.001 (2008). 18423194 PMC2732113

[b23] Peter M. E. Let-7 and miR-200 microRNAs: guardians against pluripotency and cancer progression. Cell Cycle 8, 843–852 (2009). 19221491 10.4161/cc.8.6.7907 PMC2688687

[b24] Suzuki H., Maruyama R., Yamamoto E. & Kai M. DNA methylation and microRNA dysregulation in cancer. Mol Oncol 6, 567–578, 10.1016/j.molonc.2012.07.007 (2012). 22902148 PMC5528344

[b25] Tsukamoto Y. . MicroRNA-375 is downregulated in gastric carcinomas and regulates cell survival by targeting PDK1 and 14-3-3zeta. Cancer Res 70, 2339–2349, 10.1158/0008-5472.CAN-09-2777 (2010). 20215506

[b26] Orejuela D., Jorquera R., Bergeron A., Finegold M. J. & Tanguay R. M. Hepatic stress in hereditary tyrosinemia type 1 (HT1) activates the AKT survival pathway in the fah-/- knockout mice model. J Hepatol 48, 308–317, S0168-8278(07)00590-9 (2008). 18093685 10.1016/j.jhep.2007.09.014

[b27] Josson S. . miR-409-3p/-5p promotes tumorigenesis, epithelial-to-mesenchymal transition, and bone metastasis of human prostate cancer. Clin Cancer Res 20, 4636–4646, 10.1158/1078-0432.CCR-14-0305 (2014). 24963047 PMC4155061

[b28] Josson S. . Stromal fibroblast-derived miR-409 promotes epithelial-to-mesenchymal transition and prostate tumorigenesis. Oncogene 34, 2690–2699, 10.1038/onc.2014.212 (2014). 25065597

[b29] Zheng B. . MicroRNA-409 suppresses tumour cell invasion and metastasis by directly targeting radixin in gastric cancers. Oncogene 31, 4509–4516, 10.1038/onc.2011.581 (2012). 22179828

[b30] Wan L. . MicroRNA-409-3p functions as a tumor suppressor in human lung adenocarcinoma by targeting c-Met. Cell Physiol Biochem 34, 1273–1290, 10.1159/000366337 (2014). 25278243

[b31] Hou W., Tian Q., Steuerwald N. M., Schrum L. W. & Bonkovsky H. L. The let-7 microRNA enhances heme oxygenase-1 by suppressing Bach1 and attenuates oxidant injury in human hepatocytes. Biochim Biophys Acta 1819, 1113–1122, 10.1016/j.bbagrm.2012.06.001 (2012). 22698995 PMC3480558

[b32] Bergeron A., Jorquera R., Orejuela D. & Tanguay R. M. Involvement of endoplasmic reticulum stress in hereditary tyrosinemia type I. J Biol Chem 281, 5329–5334, M506804200 (2006). 16317004 10.1074/jbc.M506804200

[b33] Murakami Y. . The progression of liver fibrosis is related with overexpression of the miR-199 and 200 families. PLoS One 6, e16081, 10.1371/journal.pone.0016081 (2011). 21283674 PMC3025920

[b34] Xiao Y. . Up-regulation of miR-200b in biliary atresia patients accelerates proliferation and migration of hepatic stallate cells by activating PI3K/Akt signaling. Cell Signal 26, 925–932, 10.1016/j.cellsig.2014.01.003 (2014). 24412919

[b35] Papadopoulos G. L., Reczko M., Simossis V. A., Sethupathy P. & Hatzigeorgiou A. G. The database of experimentally supported targets: a functional update of TarBase. Nucleic Acids Res 37, D155–158, 10.1093/nar/gkn809 (2009). 18957447 PMC2686456

[b36] Xiao F. . miRecords: an integrated resource for microRNA-target interactions. Nucleic Acids Res 37, D105–110, 10.1093/nar/gkn851 (2009). 18996891 PMC2686554

[b37] Friedman R. C., Farh K. K., Burge C. B. & Bartel D. P. Most mammalian mRNAs are conserved targets of microRNAs. Genome Res 19, 92–105, 10.1101/gr.082701.108 (2009). 18955434 PMC2612969

[b38] Mi H., Muruganujan A., Casagrande J. T. & Thomas P. D. Large-scale gene function analysis with the PANTHER classification system. Nat Protoc 8, 1551–1566, 10.1038/nprot.2013.092 (2013). 23868073 PMC6519453

[b39] Thoolen B. . Proliferative and nonproliferative lesions of the rat and mouse hepatobiliary system. Toxicol Pathol 38, 5S–81S, 10.1177/0192623310386499 (2010). 21191096

