# Supplementary material for: Identification of circulating microRNAs during the liver neoplastic process in a murine model of hereditary tyrosinemia type 1
Source: Sci Rep. 2016 Jun 10;6:27464. doi: 10.1038/srep27464 (PMC4901289; doi:10.1038/srep27464)
Supplement: Supplementary Information [file srep27464-s1.pdf]

Supplementary File S1

Identification of circulating microRNAs during the liver neoplastic process in a murine model of hereditary tyrosinemia type 1

Francesca Angileri[1,3], Geneviève Morrow[1], Jean-Yves Scoazec[2], Nicolas Gadot[2], Vincent Roy[1], Suli Huang[3,4], Tangchun Wu[3] and Robert M. Tanguay[1,*]

I. APPENDIX

A. Small RNA Library Construction

A small RNA library was generated from a customer sample using the Illumina Truseq™ Small RNA Preparation kit according to Illumina's TruSeq™ Small RNA Sample Preparation Guide[1].

B. Deep Sequencing

The purified cDNA library was used for cluster generation on Illumina's Cluster Station and then sequenced on Illumina GAIIx following vendor's instruction for running the instrument. Raw sequencing reads (40 nts) were obtained using Illumina's Sequencing Control Studio software version 2.8 (SCS v2.8) following real-time sequencing image analysis and base-calling by Illumina's Real-Time Analysis version 1.8.70 (RTA v1.8.70). The extracted sequencing reads were stored in file **[Sample]_RawData.fq** ("Sample" means "customer's sample name") and were then used in the standard data analysis, which is described in the next Section.

C. Data Analysis

A proprietary pipeline script, ACGT101-miR v4.2 (LC Sciences), was used for sequencing data analysis [2 3 4]. The flowchart of data analysis is showed in Figure 1. The key functions performed by this script and the relevant analysis results are described here:

1. Standard Data Analysis: Obtaining Mappable Sequences from Raw Reads

After the raw sequence reads, or sequenced sequences (*sequ seqs*) were extracted from image data, a series of digital filters (LC Sciences) were employed to remove various un-mappable sequencing reads:

a. Generating Unique Families of Sequ Seqs by Sorting Raw Sequencing Reads

In this step, identical *sequ seqs* in the raw data file were clustered into unique families and the file **[Sample]_unique.fq** was generated. An example of a typical entry of this file is shown below:

```
@5_18368
AGCAGAGTGGCGCAGCGGAAGCGTGCTGGGC
+
fffffefffffffffeeedeeeeceeefeee
```

where the first line indicates the *sequ seq* identification (e.g. 5_18368) composed of the index and the copy number of the *sequ seq*; the second line presents the sequence; the forth line shows the sequencing quality of the corresponding bases using the average phred score.

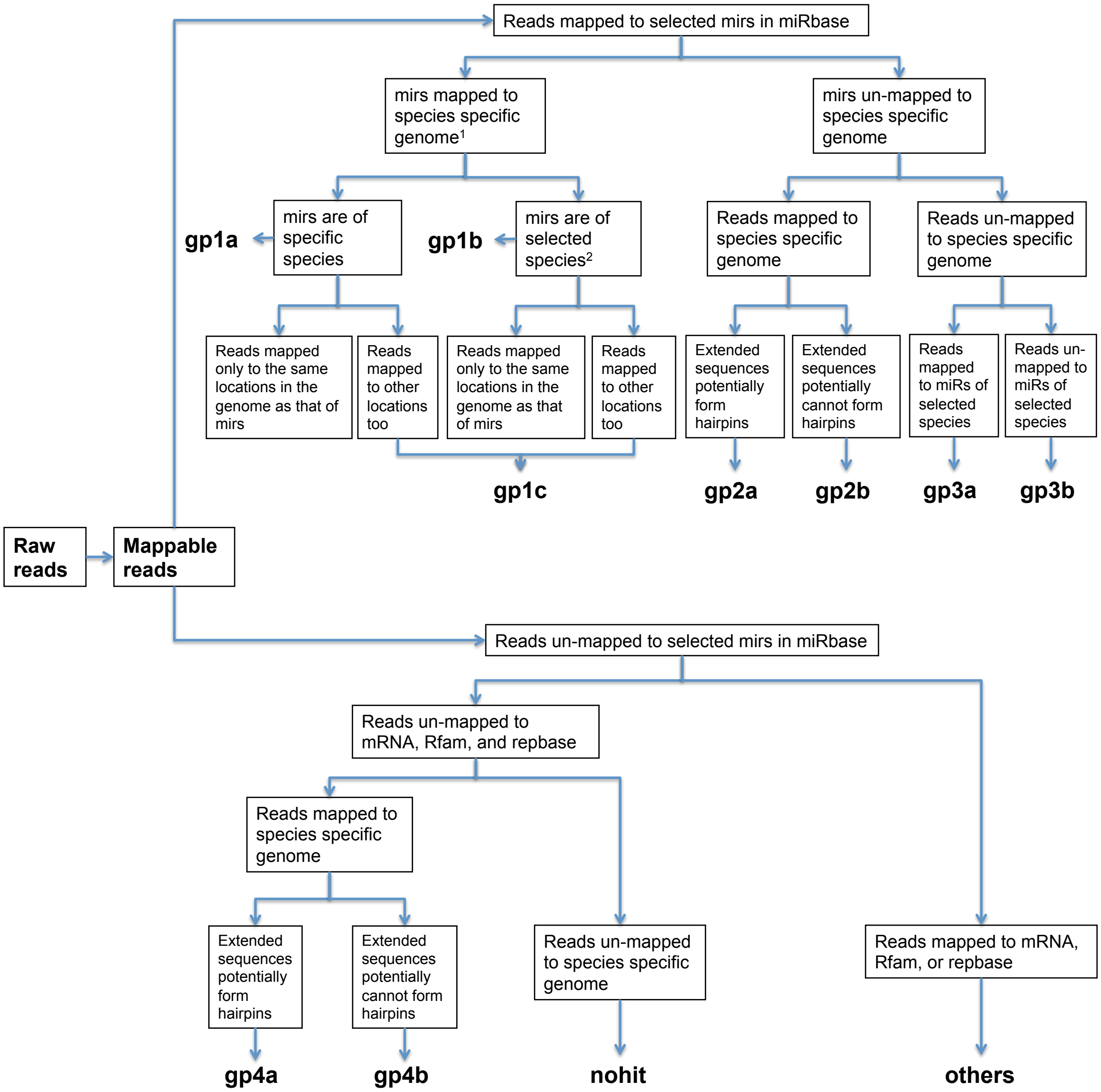

Figure 1. Flowchart of data analysis.

2575 W. Bellfort, Suite 270, Houston, Texas 77054
Tel. 713 664-7087, Fax 713 664-8181

b. Filtering Sequ Seqs

In this step, the "impurity" sequences due to sample preparation, sequencing chemistry and processes, and the optical digital resolution of the sequencer detector were removed. Those remaining *sequ seqs* (filtered *sequ seqs* with lengths between 15 and 32 bases) were grouped by families (*unique seqs*), and stored in the file **[Sample]_mappableReads.fq**, and were used to map with the reference database files.

2. In-depth Data Analysis: Mapping Mappable Reads to Reference Databases

In this Section, various "mappings" were performed on *unique seqs* against pre-miRNA (*mir*) and mature miRNA (*miR*) sequences listed in the latest release of miRBase[5, 6, 7], or genome based on the public releases of appropriate species. Mappings were also done on *mirs* of interest against genome sequence. Methods and criteria used for various mappings were documented in the ACGT-101 User's Manual[8]. Descriptions of the analyses are presented below:

a. Mapping Unique Seqs to Mirs in miRbase

The filtered *unique seqs* in **[Sample]_mappableReads.fq** were aligned against *mirs* of selected species, such as mammalian if mouse being the specified species, in miRbase. The mapped *unique seqs* were grouped as "*unique seqs* mapped to selected *mirs* in miRbase", while the remaining ones were grouped as "*unique seqs* un-mapped to selected *mirs* in miRbase".

b. Group 1

The *mir*s to which the *unique seqs* in "*unique seqs* mapped to selected *mirs* in miRbase" group were mapped were further aligned against genome of the specified species. The *mirs* mapped to the genome were sorted out and the *unique seqs* associated with these *mirs* were grouped as "*unique seqs* mapped to *mirs* that further mapped to genome". There are three sub-groups in this group,

Group 1a:

Unique seqs in this group meet the following criteria: (1) *Unique seqs* were first mapped to the selected mirs in miRbase; (2) these mirs were mapped to the genome; (3) the mirs/miRs are known mirs/miRs of the specific species. Their alignments were presented in the file **[Sample]_gp1a_aln.txt**. A summary file was also generated as **[Sample]_gp1a_sum.txt**.

Group 1b:

Unique seqs in this group meet the following criteria: (1) *Unique seqs* were mapped to the selected mirs in miRbase; (2) the mirs were mapped to the genome; (3) the mirs/miRs are known mirs/miRs of other selected species. Their alignments were presented in the file **[Sample]_gp1b_aln.txt**. A summary file was also generated as **[Sample]_gp1b_sum.txt**.

Group 1c:

This group includes *unique seqs* in Group 1a and 1b. These *unique seqs* were mapped not only to the locations of known mir/miRs but also to other locations in the genome. Their alignments were presented in the file **[Sample]_gp1c_aln.txt**. A summary file was also generated as **Sample]_gp1c_sum.txt**.

c. Group 2

Group 2a:

Unique seqs were included in Group 2a if they meet the following criteria: (1) *unique seqs* were mapped to the *mirs* of selected species; (2) the mirs were, however, not mapped to the genome of the specific species; (3) but the unique seqs were mapped to the genome; (4) <u>the extended sequences at the mapped positions of the genome potentially form hairpins.</u> Their alignments were presented in the file **[Sample]_gp2a_aln.txt**. A summary file was also generated as **[Sample]_gp2a_sum.txt**.

Group 2b:

Unique seqs were included in Group 2b if they meet the following criteria: (1) *unique seqs* were mapped to the *mirs* of selected species; (2) the mirs were, however, not mapped to the genome of the specific species; (3) but the unique seqs were mapped to the genome; (4) <u>the extended sequences at the mapped positions of the genome potentially *can NOT* form hairpins.</u> Their alignments were presented in the file **[Sample]_gp2b_aln.txt**. A summary file was also generated as **[Sample]_gp2b_sum.txt**.

d. Group 3

Group 3a:

Unique seqs were included in Group 3a if they meet the following criteria: (1) *unique seqs* were mapped to the *mirs* of selected species; (2) the mirs were, however, not mapped to the genome of specific species; (3) the *unique seqs* were also not mapped to the genome; (4) <u>but the *unique seqs* were</u>

mapped to miRs of selected species. Their alignments were presented in the file **[Sample]_gp3a_aln.txt**. A summary file was also generated as **[Sample]_gp3a_sum.txt**.

Group 3b:

Unique seqs were included in Group 3b if they meet the following criteria: (1) *unique seqs* were mapped to the *mirs* of selected species; (2) the mirs were, however, not mapped to the genome of specific species; (3) the *unique seqs* were also not mapped to the genome; (4) and the *unique seqs* were *NOT* mapped to miRs of selected species. Their alignments were presented in the file **[Sample]_gp3b_aln.txt**. A summary file was also generated as **[Sample]_gp3b_sum.txt**.

e. Mapping Unique Seqs to Selected Databases

Mappable unique seqs were mapped to other defined databases, such as mRNA, RFam, and Repbase. Custom defined databases, such as a piRNA database generated by searching Nucleotide for "piwi-interacting RNA" and "*Mus musculus*"client in NCBI, can also be searched with the mappable *unique seqs*. Mapped *unique seqs* are presented in the file **[Sample]_others.txt**.

f. Group 4

Group 4a:

Unique seqs were included in Group 4a if they meet the following criteria: (1) *unique seqs* were not mapped to the *mirs* of selected species; (2) but the *unique seqs* were mapped to the genome; (3) the extended sequences at the mapped genome positions have the propensity to form hairpins. Their alignments were presented in the file **[Sample]_gp4a_aln.txt**. A summary file was also generated as **[Sample]_gp4a_sum.txt**.

Group 4b:

Unique seqs were included in Group 4b if they meet the following criteria: (1) *unique seqs* were not mapped to the *mirs* of selected species; (2) but the *unique seqs* were mapped to the genome; (3) the extended sequences at the mapped genome positions does *NOT* have the propensity to form hairpins. Their alignments were presented in the file **[Sample]_gp4b_aln.txt**. A summary file was also generated as **[Sample]_gp4b_sum.txt**.

g. Nohit

The *unique seqs* that belongs to none of Group 1, Group 2, Group 3, Group 4, and other selected databases are defined as Nohit and presented in file **[Sample]_nohit.txt**.

3. Mapping Summary

All *unique seqs* in all groups above (excluding Group 4b and Nohit) were summarized and listed in **[Sample]_uni_miRs.txt** as mapped miRs or predicted miRs.

4. Multiple Sample Comparison

Raw reads of multiple samples are combined to generate the files **comp_unique.fq** and **comp_mappableReads.fq**. Combined reads mapped to the reference database (miRbase) are summarized in the file **comp_uni_miRs.txt** after mapping. The number of read copies from each sample is tracked during mapping and normalized for comparison. The comparison results are consolidated in the file **Report_projectID-comparison.xls**. Reads may be mapped to multiple entries of the reference database and the number of the read copies is divided by the number of mapped entries.

Normalization of sequence counts in each sample (or data set) is achieved by dividing the counts by a library size parameter of the corresponding sample. The library size parameter is a median value of the ratio between the counts a specific sample and a pseudo-reference sample. A count number in the pseudo-reference sample is the count geometric mean across all samples.

$$s_j = \underset{i}{median}\left(\frac{c_{ij}}{\left(\prod_{k=1}^{m} c_{ik}\right)^{1/m}} \right) \quad (1)$$

where, S_j is the library size parameter; c_{ij} is the count number of sequence *i* of sample *j*; *m* is the total number of samples involved

2575 W. Bellfort, Suite 270, Houston, Texas 77054
Tel. 713 664-7087, Fax 713 664-8181

Reference

---

1 TruSeq™ Small RNA Sample Preparation Guide (15004197 C), Illumina Inc., Part # 1004239 Rev. A, 2008; Catalog # RS-930-1012, Part # 15004197 Rev. B, January 2011;

2 Li, M., Xia, Y., Gu, Y., Zhang, K., Lang, Q., Chen, L., Guan, J., Luo Z., Chen, H., Li, Y., Li, Q., Li, X., Jiang, A., Shuai, S., Wang, J. , Zhu, Q., Zhou, X., Gao X., Li, X. (2010) MicroRNAome of Porcine Pre- and Postnatal Development. *PLoS ONE*, 5, e11541;

3 Wei, Z., Liu, X., Feng, T., Chang, Y., (2011) Novel and Conserved Micrornas in Dalian Purple Urchin (*Strongylocen-trotus Nudus*) Identified by Next Generation Sequencing, *International Journal of Biological Sciences*, 7, 180-192;

4 Meyer, C., Grey, F., Kreklywich, C.N., Andoh, T.F., Tirabassi, R.S., Orloff, S.L., Streblow, D.N., (2010) Cytomegalovirus MicroRNA Expression Is Tissue Specific and Is Associated with Persistence, Journal of Virology, 85, 378-89;

5 Griffiths-Jones, S., Saini, H.K., van Dongen, S., Enright, A.J., miRBase: tools for microRNA genomics, 2008, *Nucleic Acids Research*, 36, D154-D158;

6 Griffiths-Jones, S., Grocock, R.J., van Dongen, S., Bateman, A., Enright, A.J., miRBase: microRNA sequences, targets and gene nomenclature, 2006, *Nucleic Acids Research*, 34, D140-D144;

7 Griffiths-Jones, S., The microRNA Registry, 2004, *Nucleic Acids Research,* 32, D109-D111;
